# Hydrochromic carbon dots as smart sensors for water sensing in organic solvents†

**DOI:** 10.1039/c9na00493a

**Published:** 2019-10-02

**Authors:** Anitha Senthamizhan, Despina Fragouli, Brabu Balusamy, Bhushan Patil, Milan Palei, Stefania Sabella, Tamer Uyar, Athanassia Athanassiou

**Affiliations:** Smart Materials, Istituto Italiano di Tecnologia 16163 Genova Italy anitha.senthamizhan@iit.it dranitha35@gmail.com despina.fragouli@iit.it athanassia.athanassiou@iit.it; Nanoregulatory Platform, PharmaChemistry, Department of Drug Discovery and Development, Istituto Italiano di Tecnologia 16163 Genova Italy; Institute of Materials Science & Nanotechnology, Bilkent University Ankara 06800 Turkey; Department of Fiber Science and Apparel Design, College of Human Ecology, Cornell University Ithaca NY 14853 USA; Nanochemistry Department, Istituto Italiano di Tecnologia 16163 Genova Italy

## Abstract

Smart, stimuli-responsive, photoluminescent materials that undergo a visually perceptible emission color change in the presence of an external stimulus have long been attractive for use in sensor platforms. When the stimulus is the presence of water, the materials that undergo changes in their light emission properties are called hydrochromic and they can be used for the development of sensors to detect and quantify the water content in organic solvents, which is fundamental for laboratory safety and numerous industrial applications. Herein, we demonstrate the preparation of structurally different carbon dots with tunable emission wavelengths *via* a simple carbonization approach under controlled temperature and time, involving commercial brown sugar as a starting material. The detailed experimental analysis reveals the “structure-hydrochromic property” relationship of the carbon dots and assesses their capability as effective water sensors. The carbon dots that were proved most efficient for the specific application were then used to identify the presence of water in various aprotic and protic organic solvents *via* a sensing mechanism based either on the fluorescence wavelength shift or on the fluorescence intensity enhancement, respectively, attributed to the formation of intermolecular hydrogen bonds between carbon dots and water molecules. This is the first demonstration of structurally defined carbon dots in a specific application. The developed carbon dots, apart from being environmentally friendly, were proved to also be biocompatible, enabling this presented process to be a path to “green” sensors.

## Abstract 2

The present study highlights the potential of using hydrochromic carbon dots as probes for sensing water contamination in organic solvents.

## Graphical abstract

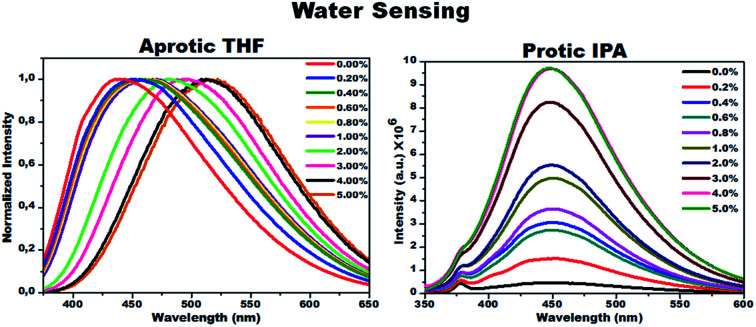

## Introduction

Visually perceptible color changes in smart stimuli-responsive materials are desirable in sensing applications. To date, enormous progress has been made in this area: solvent, vapor, light, pH and humidity-responsive materials that undergo changes in their light absorption and/or emission properties have shown great potential in practical sensing uses.^1–6^ Among the aforementioned targets, the detection and quantification of water in organic solvents has become of utmost importance for numerous industrial applications and has also attracted considerable interest in laboratory safety.^7–9^ In this context, certain chemical processes must be carefully designed and performed in order to ensure safe operation in scientific laboratories. For instance, water in Grignard reagents can create an exothermic reaction that may cause a flame. It is also well known that water in organic solvents acts as a major impurity component, causing detrimental and hazardous effects to many chemical and industrial processes. Karl-Fischer titration has already proved to be a promising analytical method for detecting water in solvents.^10^ Although the procedure is precise, it tends to be disadvantageous due to the requirement of specialized instruments, well-trained personnel, and impossible real-time on-site detection.

As an alternative, luminescent water-responsive sensors attract more and more attention, due to their simple operability, high sensitivity, on-site convenience and facile way of detecting trace amounts of water.^11–17^ Even though there are various types of luminescent sensors for humidity,^18–24^ very few systems for detecting the water content in organic solvents have been presented in the literature.^25^ These systems have certain limits since synthesis routes of such materials are usually complex, making them time-consuming and expensive. Additionally, their levels of toxicity further restrict their wide usage.

To be easily applicable for water detection in organic solvents, a luminescent water sensing material must have several important characteristics. First, it should possess high sensitivity/responsivity towards low fractions of water in organic solvents. Second, the water-induced transition of its emission color should be perceivable by the naked eye. Finally, both the synthesis of the material and the fabrication of the sensor system should be relatively simple and nonhazardous for the environment.

Fluorescent carbon dots have been proven to be promising materials for chemical and biological sensing.^26–35^ Accordingly, the preparation of multicolor emitting carbon dots has received considerable attention. It is widely acknowledged that carbon dots are composed of conjugated sp^2^ domains and surface functional groups, both contributing to their emission characteristics that can be classified into two categories: (I) excitation-dependent luminescence and (II) excitation-independent luminescence.^36–42^ For excitation-dependent luminescence, the emission modes depend on various surface defects such as C–O, C=O, O=C–OH, which can introduce different energy levels that dominate the luminescence spectra depending on the excitation energy; this kind of emission is defined as “surface state emission”. In the case of excitation-independent luminescence, the emission is attributed to the π–π* transition of the sp^2^ carbon and therefore it is called “intrinsic emission”. Specific synthetic strategies that can take advantage of these two types of emission are used for the formation of multiple color emitting carbon dots.^32–34,43–46^ Nevertheless, few studies report the systematic optimization of such carbon dots developed through carbonization.^43,47–57^ Additionally, significant research reports have shown the solvent-dependent emission of carbon dots.^58–60^ However, their hydrochromic properties are rarely discussed and for this reason, it is not known if some of these carbon dots also possess hydrochromic characteristics. Recently, two reports have proved that the carbon dots act as visual sensors of the water content in organic solvents.^61,62^ In one of the reports, Chao *et al.* observed that the emission of the carbon dots in organic solvents was gradually red shifted when the content was increased and consequently, the color change from cyan to orange was noted.^61^ In another report, the fluorescence of carbon dots was quenched in the presence of water.^62^ Nonetheless, the demonstrated hydrochromic properties of the carbon dots, descriptive insight on solvent dependent hydrochromism and the impact of structure on the hydrochromic characteristics were not provided.

In this context, we present a study on the structure-dependent hydrochromic properties of the carbon dots, which assesses their capability as effective water sensors. To evaluate this concept, we have prepared five structurally different carbon dots (CD1, CD2, CD3, CD4 and CD5), with tunable emission wavelength, in a single step using brown sugar as the only precursor material. Although all the prepared carbon dots exhibited solvatochromic characteristics, we identified differences in the hydrochromic behavior that clearly revealed the “structure-hydrochromic property” relationship of the carbon dots. Based on the structural differences of the developed carbon dots and their respective hydrochromic behaviors, we have proved that the carbon dots with dominant intrinsic emission show the most prominent hydrochromic characteristics and for this reason, they were chosen as the probes for water detection in organic solvents. To the best of our knowledge, this is the first report in which the structure of the carbon dots is correlated to their hydrochromic characteristics, and we prove that “all solvatochromic carbon dots are not mandatorily hydrochromic”. The presence of water was studied in both aprotic (tetrahydrofuran (THF), acetonitrile (ACN) and acetone) and protic solvents (isopropyl alcohol (IPA) and butyl alcohol (BuOH)). In the aprotic solvents, the presence of water was monitored by the red shift in the emission spectrum of the carbon dots, whereas in protic solvents the water detection was performed following the increase in the emission intensity of the carbon dots. Therefore, we have demonstrated for the first time that carbon dots prepared with a simple method and with specific structural characteristics can be used as efficient probes for the visual detection of water contamination in different types of solvents.

## Experimental details

### Materials

Brown sugar was purchased from the local market (Italy). All chemicals and solvents were of analytical grade (AR) and were used without further purification.

### Preparation of carbon dots

Using a simple carbonization method involving commercial brown sugar as the starting material, carbon dots were prepared. The structurally different carbon dots with tunable emission wavelength were formed in a controlled temperature and time-bound environment. Initially, 5 g of brown sugar were mixed with 5 mL of water and the mixture was carbonized in a furnace (heating rate of 5 °C min^−1^) at specific temperature and for specific time in each case, as described below, followed by cooling naturally to room temperature. As the final step, the carbonized material was dispersed in distilled water and insoluble carbon residues were filtered using a 0.2 μm filter membrane. The resultant solution was further subjected to freeze-drying to obtain carbon dots powder.

**Fig. 1:**
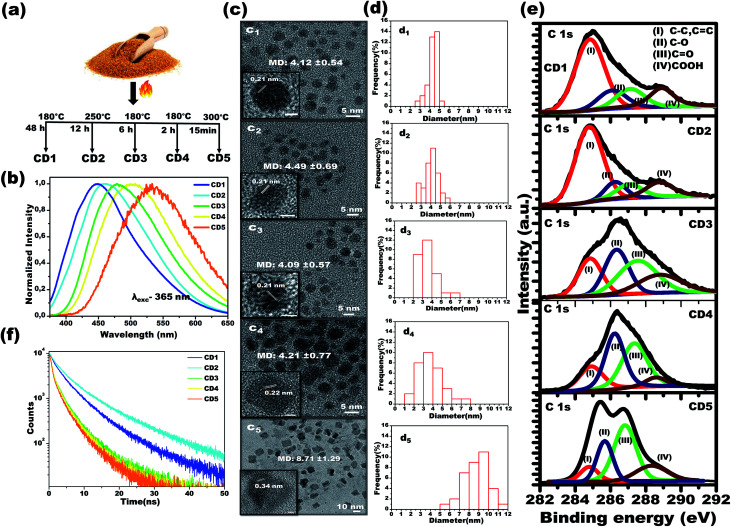
Photophysical and structural characterizations of multicolor emitting carbon dots. (a) Schematic illustration of the synthesis conditions for structurally different carbon dots, and (b) their corresponding emission spectra in water. (c) Representative HR-TEM images of carbon dots, (c_1_) CD1, (c_2_) CD2, (c_3_) CD3, (c_4_) CD4 and (c_5_) CD5. Inset panels show the lattice spacing of a single carbon dot. (d) Size distribution histograms of carbon dots (d_1_) CD1 (d_2_) CD2 (d_3_) CD3 (d_4_) CD4 and (d_5_) CD5. (e) Representative high-resolution XPS spectra of carbon dots. Deconvoluted XPS spectra of carbon (C 1s) fitted with four components: I- (sp^2^ C (C–C, C=C)), II- (sp^3^ C (C–O)), III- (C=O), and IV- (COOH). (f) Photoluminescence lifetime decay curves of the carbon dots.

Carbon dots 1 (CD1) were produced upon heating the sugar/water mixture at 180 °C, for 48 h, and upon excitation at 365 nm, the emission spectrum of their aqueous solution (2.5 mg mL^−1^) was centered at 448 nm. Carbon dots 2 (CD2) were produced upon heating the sugar/water mixture at 250 °C, for 12 h, and upon excitation at 365 nm, the emission spectrum of their aqueous solution (2.5 mg mL^−1^) was centered at 462 nm. Carbon dots 3 (CD3) were produced upon heating the sugar/water mixture at 180 °C, for 6 h and upon excitation at 365 nm, the emission spectrum of their aqueous solution was centered at 483 nm. Carbon dots 4 (CD4) were produced upon heating the brown sugar/water mixture at 180 °C, for 2 h and upon excitation at 365 nm, the emission spectrum of their aqueous solution (2.5 mg mL^−1^) was centered at 504 nm. Finally, carbon dots 5 (CD5) were produced upon heating the sugar/water mixture at 300 °C, for 15 min, and upon excitation at 365 nm, the emission spectrum of their aqueous solution (2.5 mg mL^−1^) was centered at 532 nm. See Fig. 1a.

### Sensing experiments for identifying the water content in organic solvents

Different concentrations (0.2% to 5.0%, v/v) of hydrated solvents were prepared by the careful addition of the required amount of water into the solvents. The carbon dots (2 mg mL^−1^) were allowed to react with the hydrated solvents for a period of one hour. The change in the emission wavelength was studied under exposure to UV light (*λ*_max_ – 365 nm) and the fluorescence spectra were recorded.

### Cell culture and treatment

Caco-2 human colon carcinoma cells were maintained in complete Dulbecco's Modified Eagle's Medium (DMEM, Thermo Fisher Scientific) supplemented with 10% (v/v) FBS (Hyclone) and 1% v/v penicillin/streptomycin (EuroClone) and 1% v/v MEM non-essential amino acids solution (NEAA, Thermo Fisher Scientific) at 37 °C under a humidified controlled atmosphere with a 95% to 5% ratio of air/CO_2_. The cells were seeded at the density of 1 × 10^5^ cells per mL in 96-well plates and incubated for 24 h prior to the exposure to carbon dots. All the treatments were performed in complete cell culture medium followed by dilution of different carbon dots, *i.e.*, CD1, CD2, CD3, CD4 and CD5 at a final concentration of 100 and 250 μg mL^−1^. They were further incubated for a period of 24 and 48 h in three replicates, then biocompatibility evaluations were performed using WST-1 assay (cell viability) and H_2_DCF-DA assay (intracellular oxidative stress).

### WST-1 cell viability assay

The metabolic activity of the respective carbon dots-exposed cells were measured using a colorimetric nonradioactive WST-1 assay (Roche) according to the instructions provided by the manufacturer. In brief, cells were gently washed three times with phosphate buffered saline (PBS) after specified treatment points at the indicated concentrations and constituted by cell culture medium. Further, 10 μL of WST-1 reagent were added to each well and incubated under a humidified atmosphere of 5% CO_2_ at 37 °C for 3 h. The formazan compound produced by mitochondrial dehydrogenase from metabolically intact cells was quantified at 440 nm using a microplate reader (Infinite M200, Tecan) and the absorbance was compared to the untreated control.

The percentage of cell viability was determined using the following equation:% cell viability = (absorbance treated/absorbance control) × 100%,and the data were expressed as mean ± SD of three replicates.

### H_2_DCF-DA assay

Intracellular oxidative stress was measured using cell permeant, 2′,7′-dichlorodihydrofluorescein diacetate dye (H_2_DCF-DA, Thermo Fisher Scientific), which was decayed by cellular esterases to a non-fluorescent compound and further oxidized into highly fluorescent dichlorofluorescein (DCF). Following the specific incubation period, the cells were washed using Hank's Balanced Salt Solution (HBSS) and stained with 5 μM H_2_DCF-DA dye for 45 min at 37 °C, then washed and loaded with HBSS. Further intracellular reactive oxygen species (ROS) generation was measured using a microplate reader (Infinite M200, Tecan) with the temperature maintained at 37 °C by setting excitation and emission filters at 480 and 520 nm, respectively. The results were expressed as mean ± SD of three replicates by normalizing the values with respect to negative controls (expressed as 100%). The cells exposed to 5 mM H_2_O_2_ for induction of oxidative stress served as the positive control.

### Characterization

The morphology and size of the carbon dots were measured using transmission electron microscopy (TEM, Tecnai G2 F30 (Oregon, USA)). The functional groups present in the carbon dots were studied using a Fourier Transform Infrared (FTIR) spectrometer (Equinox 70 FT-IR, Bruker) coupled to an attenuated total reflectance (ATR) accessory (MIRacle ATR, PIKE Technologies). The chemical composition of the carbon dots was studied using X-ray photoelectron spectroscopy (XPS, Thermo K-alpha-monochromatic). Fluorescence emission spectra were measured by a time-resolved fluorescence spectrophotometer (FL-1057 TCSPC, NJ, USA). ^1^H-NMR spectra were recorded at 400 MHz (Bruker DPX-400) and analysis was done by using Mestrenova software. Time-resolved PL emission spectra were taken using an FLS920 spectrofluorometer from Edinburgh Instruments. Time decay profiles were taken using a diode laser of pulse width 50 ps and a repetition rate of 0.05–1.00 MHz in order to ensure the complete decay of emission between the excitation pulses. XRD spectra were collected using a PANalytical Empyrean X-ray diffractometer equipped with a 1.8 kW Cu Kα ceramic X-ray tube (*λ* = 1.5418 Å).

## Results and discussion

### Characterization of the developed carbon dots

In order to attain structurally different carbon dots through the carbonization process, both temperature and treatment time of the brown sugar were tuned, and the optimized conditions are reported in Fig. 1a. Under a single excitation wavelength of 365 nm, the aqueous dispersions of the various prepared carbon dots showed distinctively different emission spectra: CD1 centered at 448 nm, CD2 centered at 462 nm, CD3 centered at 483 nm, CD4 centered at 504 nm and CD5 centered at 532 nm, as depicted in Fig. 1b. It is interesting to note that the emission spectra of carbon dots shift from 504 nm to 448 nm with increasing carbonization time from 2 h to 48 h at 180 °C as noted in the spectra in Fig. 1b. However, the CD2 and CD5 were formed at the elevated temperatures of 250 °C and 300 °C for a duration of 12 h and 15 min, respectively. The photoluminescence quantum yields of the different emitting carbon dots were calculated to be 5.6 ± 0.5%, 7 ± 0.4%, 3.5 ± 0.3%, 1.5 ± 0.4% and 1 ± 0.3% for CD1, CD2, CD3, CD4 and CD5, respectively. The attained results indicate that the carbonization degree has obvious effects on the emission characteristics of carbon dots.

To investigate the underlying mechanism of the emission of carbon dots in water, studies on their size, shape and surface functional groups were performed and are discussed in the following sections. Fig. 1c shows the high-resolution transmission electron microscopy (HRTEM) images of CD1, CD2, CD3 and CD4, illustrating the spherical shape with average size (refer to Fig. 1d) of 4.12 ± 0.54, 4.49 ± 0.69, 4.09 ± 0.57 and 4.21 ± 0.77 nm, respectively. An exception to this is CD5, which appears irregular in shape with an average size of 8.71 ± 1.29. In addition, two types of lattice fringe distances have been noticed at 0.21 nm (CD1 to CD4) and 0.34 nm (CD5). These can be attributed to the *d* spacing of the graphene (100) and graphite (001) planes, respectively.^43,63^ Since no important differences were observed between the sizes of the carbon dots (CD1, CD2, CD3 and CD4), the variation in the fluorescence cannot be directly correlated to the size and shape of the carbon dots, but rather to different surface defects.

The characteristic peaks of C=O, O–H, C–H and C–O, C=C vibration modes of the carbon dots^51^ have been affirmed by the FTIR spectra as depicted in Fig. S1,† with no significant differences in the peak positions between the different carbon dots. In order to attain further information on the surface functional groups and the definite composition of each carbon dot type, surface-sensitive XPS measurements were performed. Strong signals of C 1s and O 1s are shown in Fig. S2a† and their atomic percentages are tabulated in Fig. S2b,† confirming the absence of impurities. The high-resolution C 1s XPS spectra are displayed in Fig. 1e and the following contributions were assigned: carbon sp^2^ (C–C, C=C, I), carbon sp^3^ (C–O, II), carbonyl carbons (C=O, III), and carboxyl carbons (COOH, IV).^37–39,45^

The X-ray diffraction (XRD) patterns of the carbon dots powder in Fig. S3† demonstrate a broad single diffraction peak centered at ∼22° (*d* = 0.40 nm), ∼23° (*d* = 0.38 nm), ∼21.8° (*d* = 0.40 nm), ∼20.7° (*d* = 0.43 nm) and ∼22.8° (*d* = 0.39 nm) for the CD1, CD2, CD3, CD4 and CD5, respectively. In all cases, the attained *d* spacing values are slightly higher than that of graphite (*d* = 0.34 nm) along the (002) direction, and this can be attributed to the enlarged interlayer distance resulting from the presence of oxygenated functional groups on the surface of the carbon dots. The combined results of HRTEM and XRD indicate that the prepared carbon dots have both the (100) in-plane lattice spacing and the (002) interlayer spacing of graphite.^64,65^ The outcome affirms the presence of oxygenated functional groups consisting of hydroxyl, carboxyl and carbonyl groups on the surface of the carbon cores in the carbon dots. As noted in the spectra, the percentage area of sp^2^ C bonding is the highest for the CD1 and the CD2, whereas, for the rest of the carbon dots, oxygenated surface functional groups became dominant. Therefore, the prolonged thermal treatment during the CD1 formation (180 °C, 48 h) has led to a higher carbonization degree, emanating less oxygen-related surface groups. In the CD5, the alcoholic carbon species are prevalent, indicating that 15 minutes of treatment of the brown sugar at a high temperature of 300 °C is enough for the formation of an efficient number of surface defects. The observed results indicate that the structure of the carbon dots strongly depends on the carbonization degree of sugar, leading to the tunable fluorescence.

To gain more insight into the effect of the π conjugated domain and surface states, 2D excitation-emission contour maps (Fig. S4†) were recorded. It was found that CD3, CD4, and CD5 exhibited strong excitation-dependent emission as compared to CD1 and CD2. All the spectra confirmed that the CD3, CD4, and CD5, the ones formed after thermal treatment for short times, have more oxygenated surface and exhibit strong excitation-dependent emission, attributed to different surface states like C–O, C=O, O=C–OH with specific energies, as clearly shown in the XPS spectra. On the other hand, the prolonged thermal treatment leads to the reduction of oxygenated functional groups in CD1, which exhibit excitation-independent emission of the excitation wavelength (250–360 nm) that originated from the intrinsic state of the sp^2^ domain.^36–42^ The oxygenated surface functional groups can serve as trap states for excitons and this would benefit the surface-state-related fluorescence.^36–40^ The significant increase in trap states also favors the rapid decay of excitons following non-radiative channels, which results in shortened fluorescence lifetimes (Fig. 1f). The average fluorescence lifetime was calculated to be 6.02, 8.03, 3.78, 2.94 and 2.64 ns for CD1, CD2, CD3, CD4 and CD5, respectively.

**Fig. 2:**
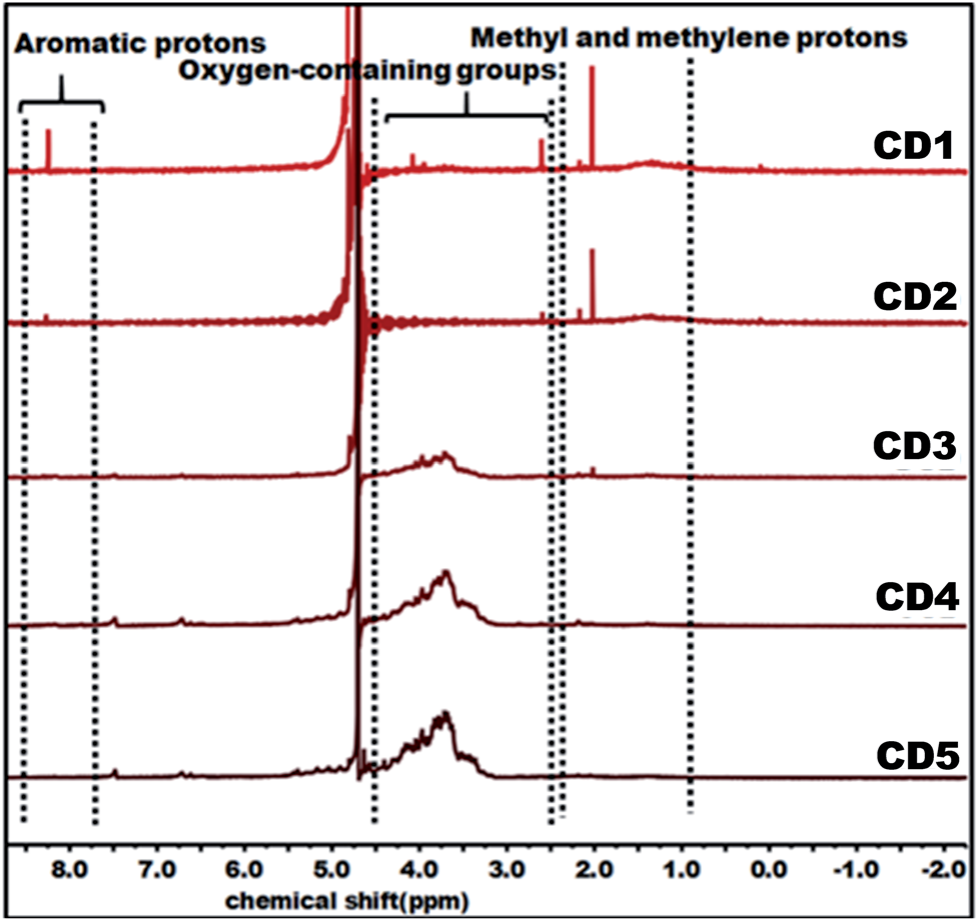
^1^H NMR (D_2_O) spectra of carbon dots.

The structural variation of carbon dots was further corroborated by ^1^H NMR as depicted in Fig. 2. In the obtained spectra, peaks in the range of 0.8–2.2, 2.5–4.5, and 8.0–9.0 ppm were ascribed to methyl and methylene protons, protons in the vicinity of oxygen-containing groups (hydroxyl and carbonyl), and aromatic ring hydrogen, respectively.^66–68^ The observed strong signal at 8.0–9.0 ppm intensely supports that CD1 and CD2 consist of an aromatic carbon-rich architecture. In addition, the peaks in the range of 3.0–4.5 ppm were significantly reduced as compared with CD3, CD4, and CD5 and this might be ascribed to the lower quantity of oxygen-containing groups in both cases. The observed small peak at 1.37 ppm is attributed to the methylene groups of the aliphatic chain and the intense peak at 2.18 represents the methyl ketone. As evident in the spectra, the peaks for CD1 and CD2 are nearly identical, indicating their structural similarity. In the case of CD4 and CD5, there is no detectable signal that corresponds to aromatic ring hydrogens and the presence of abundant oxygen-containing groups were verified through the strong signal detected in the range of 3.0–4.5 ppm. Overall, the observed results reveal the significant difference in the structural features of the obtained carbon dots, in terms of aromaticity and surface oxygen-containing functional groups that are expected to significantly affect the hydrochromic emission properties.

The adverse effects of new nanomaterials on human health and environmental contamination must be taken into consideration in the development of these innovative materials. To offer an initial indication of the potential biological impact of carbon dots, a biocompatibility profile, namely cell viability and intracellular oxidative stress, was measured using Caco-2 human colon carcinoma cells, representing epithelial intestinal cells. The WST-1 assay showed that cells exposed to increasing doses of carbon dots for 24 and 48 h did not elicit a significant reduction in cellular viability (Fig. S5†). Further, results obtained from H_2_DCF-DA, an assay that enables the measurements of intracellular reactive oxygen species (ROS), demonstrated no detectable oxidative stress upon cell treatment with carbon dots. Overall, these data provide a preliminary indication that carbon dots are biocompatible materials within the studied dose and exposure regime.

### Hydrochromic emission of carbon dots and water sensing in organic solvents

**Fig. 3:**
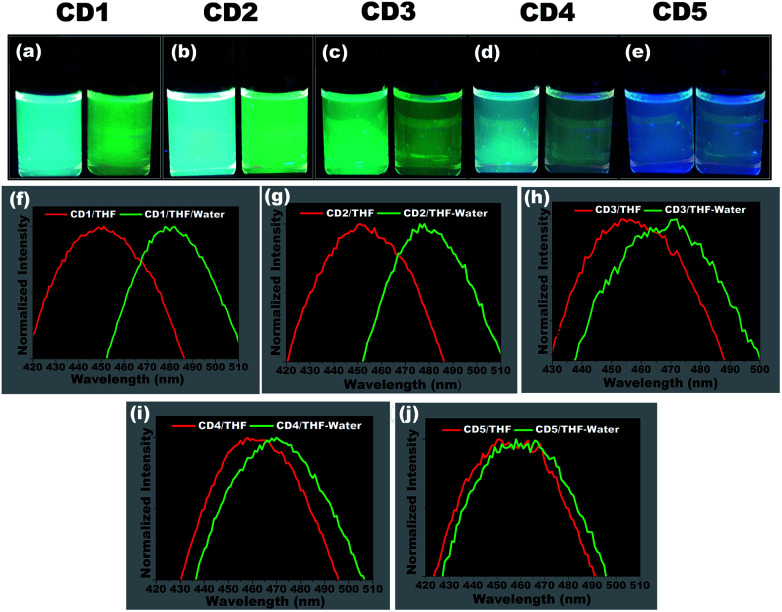
Demonstration and screening of suitable hydrochromic carbon dots. (a–e) Visual differences in the emission of carbon dots in THF (left) and THF/water (50 : 50 (% v/v)) (right). The photographs were taken immediately after adding the water to THF under UV light (*λ*_max_ – 365 nm). (a) CD1, (b) CD2, (c) CD3, (d) CD4, (e) CD5 and (f–j) their corresponding photoluminescence spectra under an excitation wavelength of 365 nm. The expanded view of the emission peak clearly demonstrates the water-induced peak shift in all carbon dots upon the addition of water. CD1 and CD2 showed the most prominent fluorescence difference, indicating their hydrochromic behavior.

To study the hydrochromic emission behavior of the formed carbon dots, initially the fluorescence emission was examined under UV light (*λ*_max_ – 365 nm) when they were suspended in the aprotic solvent THF and in THF/water (50 : 50 (% v/v)) (photographs in Fig. 3a–e). It was observed that the different carbon dots responded differently to the presence of water. The CD1 and CD2 are the ones that show the most prominent hydrochromic behavior with a fluorescence shift from 447 nm to 481 nm (Fig. 3f) and 452 nm to 480 nm (Fig. 3g), respectively, and visually noticeable emission color change from blue to green under UV light (*λ*_max_ – 365 nm). On the other hand, CD3 and CD4 show a shift in the presence of water in THF from 455 nm to 468 nm (Fig. 3h) and 462 nm to 472 m (Fig. 3i), respectively. Finally, CD5 did not demonstrate such a hydrochromic effect under the same conditions, since the variation in the emission spectrum is not so prominent in the presence of water in THF as observed in Fig. 3j. Based on the structural differences in the carbon dots and their respective hydrochromic behaviors, we reasoned that the carbon dots with dominant π character would show prominent hydrochromic emission. Taking into account the low hydrochromism of CD5, it is straightforward to conclude that the water-promoted hydrogen bonded network is itself not sufficient to induce the fluorescence changes in carbon dots.^69–71^

**Fig. 4:**
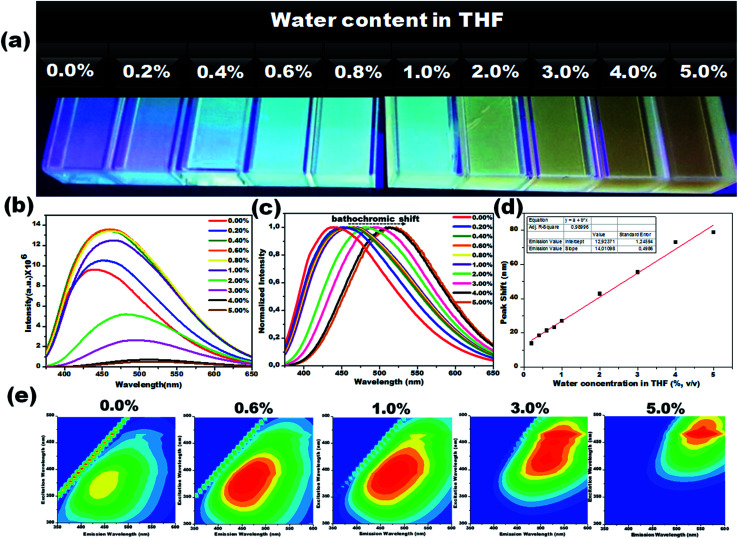
Visual colorimetric water sensing performance of hydrated aprotic THF. (a) Photograph of carbon dots in THF and hydrated THF with varied the water content (0.2% to 5.0%, v/v) under UV light (*λ*_max_ – 365 nm). The corresponding photoluminescence spectra (b) and their normalized photoluminescence spectra (c). (d) Emission peak shift *vs.* water content in THF. A significant bathochromic-shift from 439 to 518 nm was noted in the emission spectra when the water content increased from 0.2% to 5.0%, v/v. (e) Representative 2D excitation-emission contour map of CD1 in THF and THF with different water concentrations.

Due to their prominent hydrochromic effect, the CD1 were selected for an in-depth demonstration of sensing of small amounts of water in organic solvents, starting from the aprotic THF. The water sensing performance of CD1 in hydrated THF with small water contents under UV light (*λ*_max_ – 365 nm) is presented in Fig. 4. The addition of protic water induces the hydrochromic effect, leading to a clear gradual red shift in the emission spectra. All the emission spectra were taken one hour after the addition of water (nevertheless, the hydrochromic effect is visible even when the spectra are recorded immediately after the water addition, as with the ones presented in Fig. S6 and Video S1†). As a control, pure THF was gradually added to the initial THF solution and no color emission was evident (Fig. S7†). Furthermore, it is interesting to note that there were no significant changes in the emission color of CD1/THF (2 mg mL^−1^) when IPA (2 mL) was introduced, thus proving their selective response towards water (see Fig. S8 and Video S2†).

The emission wavelength transition behavior of carbon dots (CD1/THF, 2 mg mL^−1^) at 365 nm excitation began from the lowest water concentration (0.2%, v/v), and continued with a strong shift in the emission wavelength (439 to 518 nm) on going from pure THF to THF mixed with the highest water concentration used (5.0%, v/v), spotlighting the remarkable sensing ability of the system, even by the naked eye (see the photographs in Fig. 4a–c). Above 5.0% v/v water addition, the emission wavelength does not show any further prominent shift. The dependence of the fluorescence emission on the different concentrations of water in THF is plotted in Fig. 4d. A linear relationship of emission wavelength *versus* water content can be established, which proves that the carbon dots can be a quantitative PL sensor for traces of water in THF. The representative 2D excitation-emission contour maps are depicted in Fig. 4e. The gradual shift in the emission peak was observed upon increasing the hydration rate as was previously observed in PL measurement. The detailed analysis of the spectra has shown that the variations in the emission intensity are not linearly related to the water content (Fig. 4b). At lower water concentrations (0.2–0.8%, v/v) in THF, apart from the red shift in the fluorescence of the CD1, an intensity enhancement was also observed. At higher water concentrations, the red shift continued but the intensity of the emission decreased (Fig. 4b). We propose that in aprotic solvents like THF, which are not hydrogen donors, the addition of low concentrations of water induces a hydrogen-bonded network between the carbon dots that restricts non-radiative paths like intramolecular rotation, and rigidifies the structure of the carbon dots, resulting in the enhancement of fluorescence. Further increase of the water content in THF results in the decrease of the emission intensity along with a red shift in the emission maximum. The red shift can be connected to the continuous aggregation of the carbon dots, whereas the intensity decrease can be attributed to the increased non-radiative relaxation pathways through the enhanced vibronic coupling of the carbon dots with the surrounding water molecules, or carbon dots.

**Fig. 5:**
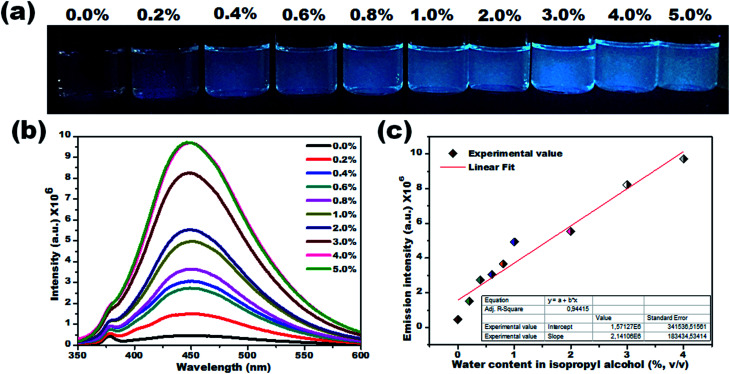
Visual colorimetric water sensing performance of CD1 on hydrated protic isopropyl alcohol. (a) Photograph of CD1 in IPA and hydrated IPA with varied water content (0.2% to 5.0% v/v) under UV light (*λ*_max_ – 365 nm), and (b) their corresponding PL emission spectra (*λ*_ex_ – 330 nm). The gradual enhancement in the emission intensity was noted upon increasing the water content in isopropyl alcohol. (c) Calibration curve (percentage of water content *vs.* emission intensity).

To verify this, the PL lifetime decay curves of CD1/THF before and after adding water were recorded and are depicted in Fig. S9.† The average lifetime increased from 4.7 ns to 7.4 ns for the solution of CD1/THF when water was added, and this observation proved the enhancement of the nonradiative relaxation channel through the presence of intermolecular hydrogen bonding interactions between the carbon dots and water molecules. Next, the efficiency of the developed CD1 for the detection of water in a model protic solvent, IPA, (Fig. 5a–c) was studied under the same conditions as described in Fig. 4 for the water content evaluation in THF. In contrast to the behavior of the carbon dots in the hydrated aprotic THF, where they showed a shift in their emission wavelength, in the hydrated protic IPA, they showed gradual fluorescence intensity enhancement with the increase of the water fraction, along with a slight blue shift (450 nm to 446 nm) as depicted in Fig. S10 and S11.† A linear relationship exists between the maximum emission intensity and the water content, affirming the possibility for the quantitative analysis of the water content also in IPA. It can be assumed that in the presence of protic solvents made up of hydrogen bond donating molecules, the carbon dots already form intermolecular hydrogen bonds with the surrounding solvent.^11,72,73^ This binding with IPA seems to create favorable non-radiative relaxation pathways since the CD1 photoluminescence in this solvent is low. The addition of small amounts of water, up to 4.0% is proven to induce an enhancement in the emission intensity without really affecting the emission wavelength; thus, we can safely say that the radiative pathway of the intrinsic core photoluminescence emission after the photoexcitation prevails over the non-radiative paths in the presence of water. Non-radiative paths are considered to be the vibronic coupling of the dots with the surrounding molecules, vibrational mode excitation, intramolecular rotation, *etc.*; all are known to strongly quench the photoluminescence emission.^74–77^ Since water is the smallest protic solvent, it is expected to reduce the vibronic coupling non-emitting pathways when interacting with the carbon dots.

**Fig. 6:**
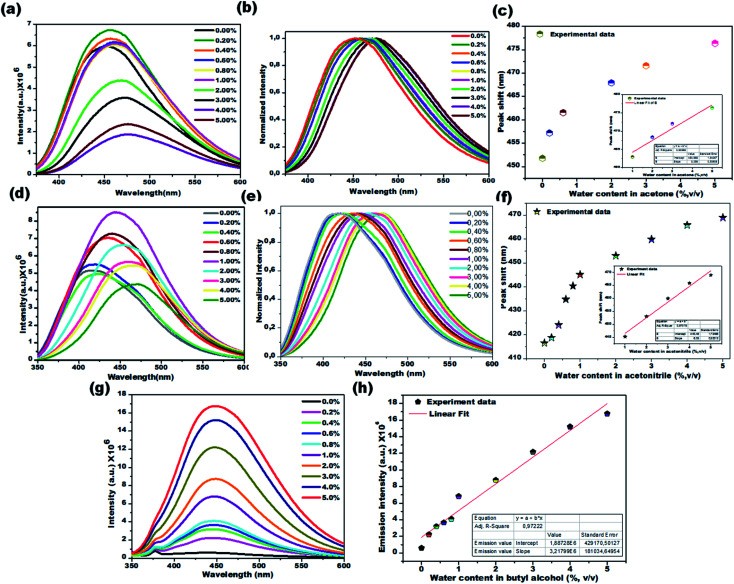
Colorimetric detection of water in acetone (a–c) acetonitrile (d–f), and butyl alcohol (g and h). PL emission spectra of CD1 on varying the water content from 0.2% to 5.0% v/v (emission spectra-a, d and g: normalized emission spectra: b and e) and their calibration curve (percentage of water content *vs.* peak shift, c, f and h).

Water sensing experiments were extended to various aprotic and protic solvents and they resulted in different responses upon exposure of the CD1 to the hydrated solvents for one hour, clearly depicted in Fig. 6a–c (aprotic acetone), Fig. 6d–f (aprotic acetonitrile) and Fig. 6g and h (protic BuOH). The spectral analysis of the CD1 in the corresponding solvents revealed a clear visual change in the fluorescence emission wavelength for the aprotic solvents acetone (451 to 476 nm) and MeCN (416 to 468 nm), and an enhancement in the fluorescence intensity for the protic solvent BuOH in the presence of water, in agreement with the above observations for the THF and IPA, respectively. The observed differences in the sensing performance can be attributed to the ability of water to make hydrogen bond interactions with carbon dots in the presence of either a protic or aprotic solvent environment.^11–13^ The above observations indicate that the sensing capability of carbon dots is correlated to the spectral shift in aprotic solvents and fluorescence intensity enhancement in protic solvents.

In comparison to other reported luminescence-based water sensing processes (please refer to ESI Table 1†), the proposed technique proves to be more straightforward and cost-effective, thus facilitating the preparation of highly sensitive hydrochromic biocompatible carbon dots. Our study demonstrates a highly desirable solvent specific visual colorimetric transition response even at low concentrations (0.2%, v/v), opening new avenues towards designing a sensor system for the detection of hydrated solvents.

## Conclusions

We have demonstrated the feasibility of preparing structurally different carbon dots with tunable emission wavelength through carbonization. This study has highlighted the potential for using hydrochromic carbon dots as probes for sensing water contamination in organic solvents. This is the first report wherein structurally tuned carbon dots demonstrate solvent specific hydrochromic characteristics. Overall, the proposed fluorescent carbon dots (I) display the structure-hydrochromic property relationship, (II) prove that “all solvatochromic carbon dots are not mandatorily hydrochromic, (III) show a red-shift in their fluorescence upon hydration of aprotic solvents, which is visible to the naked eye, (IV) show an increase in their fluorescence intensity upon hydration of protic solvents, which is visible to the naked eye, (V) are produced in a simple way with the possibility of scaling up, and are biocompatible and low-cost. This work paves the way for a new class of carbon dots with enhanced properties, possessing additional functionalities and water sensing performances. Although water sensing is used to prove this concept, this approach can be generalized to various applications including humidity sensing, mapping of sweat pores and safety controls, among others.

## Conflicts of interest

There are no conflicts to declare.

## Supplementary Material

NA-001-C9NA00493A-s001

NA-001-C9NA00493A-s002

NA-001-C9NA00493A-s003
