# Supplementary material for: Hydrochromic carbon dots as smart sensors for water sensing in organic solvents
Source: Nanoscale Adv. 2019 Oct 2;1(11):4258–67. doi: 10.1039/c9na00493a (PMC9419604; doi:10.1039/c9na00493a)
Supplement: NA-001-C9NA00493A-s001 [file NA-001-C9NA00493A-s001.pdf]

Please do not adjust margins

Nanoscale Advances

Supplementary Information

Hydrochromic carbon dots as smart sensors for water sensing in organic solvents

Anitha Senthamizhan*[a], Despina Fragouli*[a], Brabu Balusamy[b], Bhushan Patil[c], Milan Palei[e], Stefania Sabella[b], Tamer Uyar[c,d] and Athanassia Athanassiou*[a]

[a] *Smart Materials, Istituto Italiano di Tecnologia, 16163 Genova, Italy.* anitha.senthamizhan@iit.it, dranitha35@gmail.com, despina.fragouli@iit.it, athanassia.athanassiou@iit.it
[b] *Nanoregulatory Platform, PharmaChemistry, Department of Drug Discovery and Development, Istituto Italiano di Tecnologia, 16163 Genova, Italy*
[c] *Institute of Materials Science & Nanotechnology, Bilkent University, Ankara, 06800, Turkey*
[d] *Department of Fiber Science and Apparel Design, College of Human Ecology, Cornell University, Ithaca, NY, 14853, USA*
[e] *Nanochemistry Department, Istituto Italiano di Tecnologia, 16163 Genova, Italy*

Please do not adjust margins

Please do not adjust margins

Table of contents

Figure No	Description	Page No
S1	Fourier-transform infrared spectra of the carbon dots	3
S2	XPS survey spectra of carbon dots	4
S3	XRD spectra of carbon dots	5
S4	Representative 2D excitation-emission contour map of carbon dots	6
S5	Biocompatibility studies of carbon dots	7
S6	Visual colorimetric changes in the emission of CD1 in THF upon addition of water	8
S7	Visual colorimetric changes in the emission of CD1 in THF upon addition of pure THF	8
S8	Selective water sensing performance of CD1/THF	9
S9	Time-resolved photoluminescence decay curves	9
S10	Excitation-emission contour maps of carbon dots in IPA and hydrated IPA	10
S11	Colorimetric sensing performance of hydrated Isopropyl alcohol	11

Supplementary Table

Supplementary Table 1: Comparison of the carbon dot based photoluminescent water sensor with other works.

Supplementary Movie

Supplementary Movie 1: Demonstration of water sensing performance. An immediate distinguishable color change of CD1 is noticed upon exposure to water.

Supplementary Movie 2: Demonstration of selective water sensing performance. The IPA, as an example of polar protic solvent is introduced into CD1/THF (2 mL) to evaluate the selective sensing response towards water. There is no significant change in the emission color of the CD1.

This journal is © The Royal Society of Chemistry 20xx

Please do not adjust margins

Please do not adjust margins

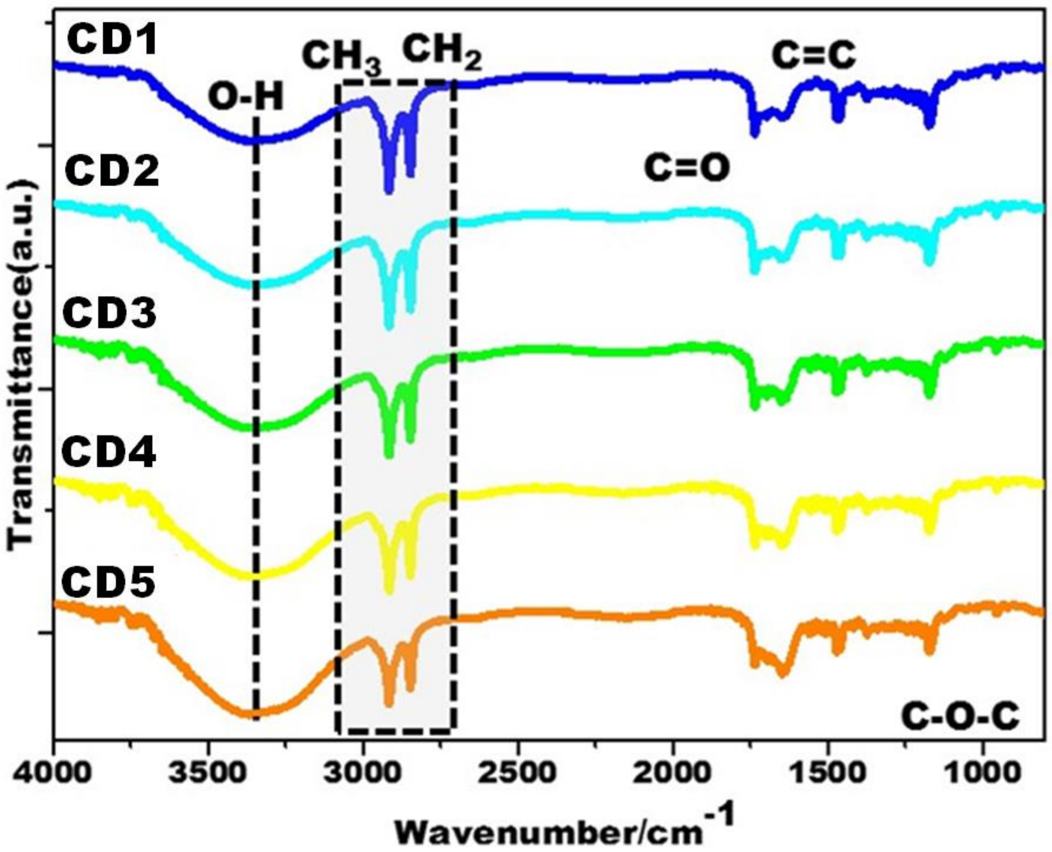

Fig. S1 Fourier-transform infrared spectra of the carbon dots.

Please do not adjust margins

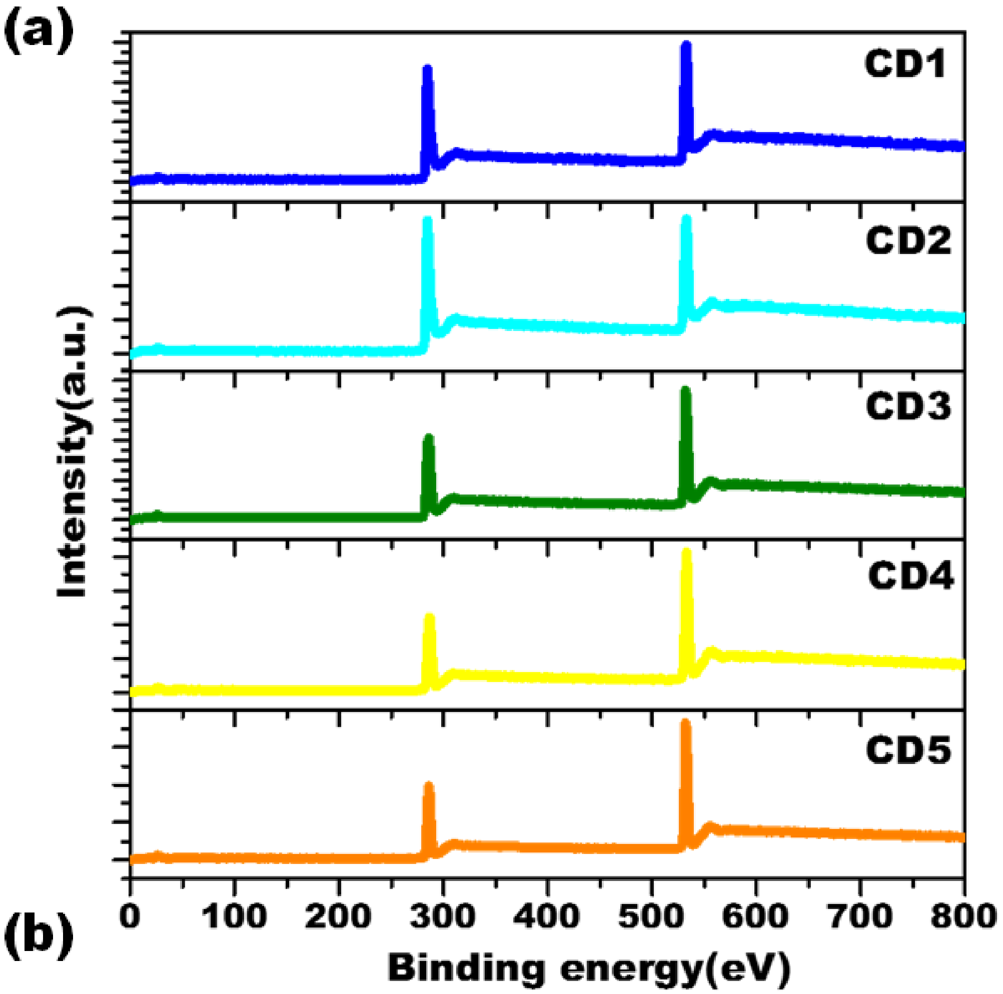

(b)

Samples	Atomic concentration (%)	
	C	O
CD1	71.24	28.76
CD2	73.79	26.21
CD3	66.98	33.02
CD4	60.73	39.27
CD5	61.41	38.59

Fig. S2 XPS survey spectra of carbon dots (a) and the table (b) containing atomic concentration of present elements (%).

This journal is © The Royal Society of Chemistry 20xx

Please do not adjust margins

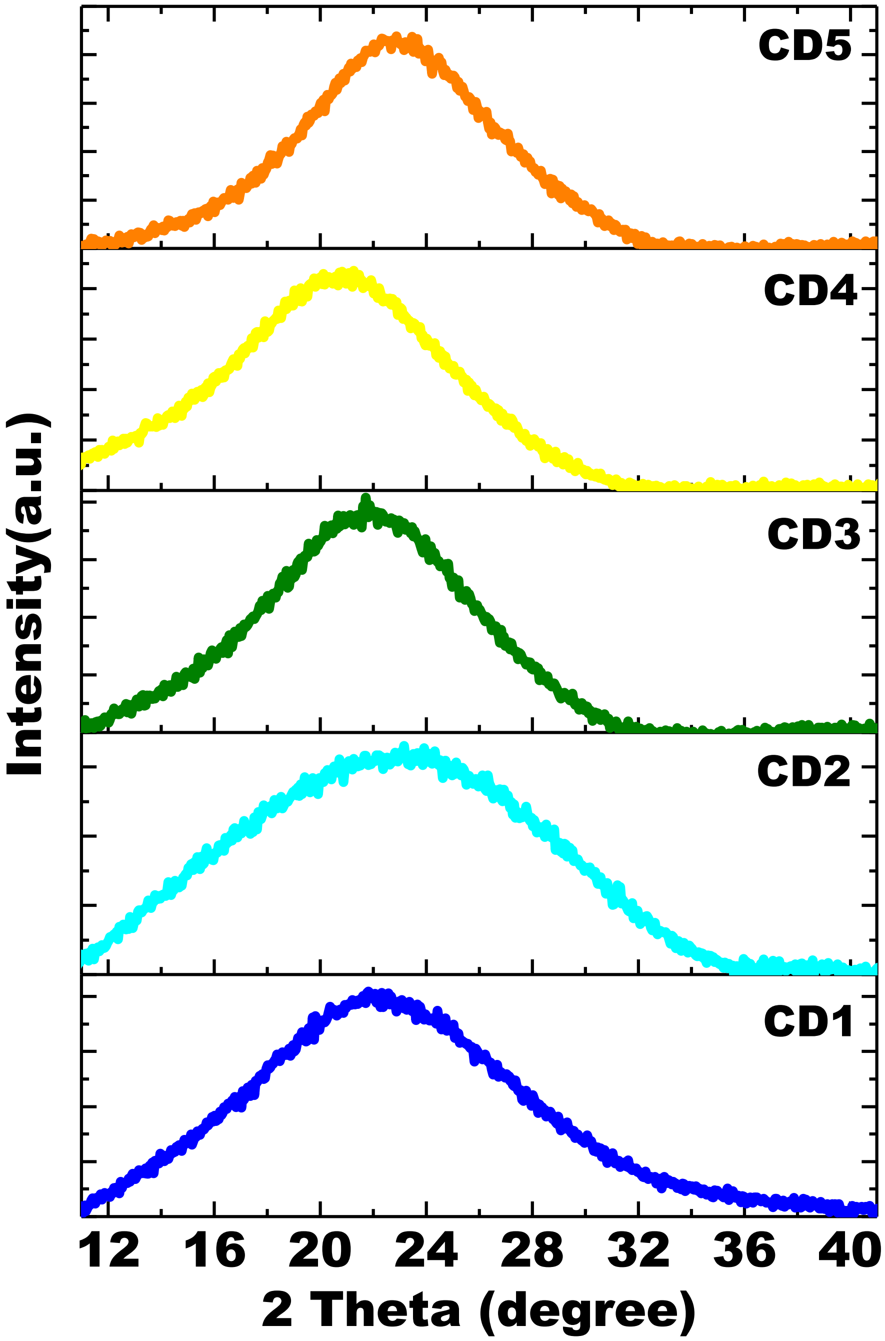

Fig. S3 XRD spectra of carbon dots.

Please do not adjust margins

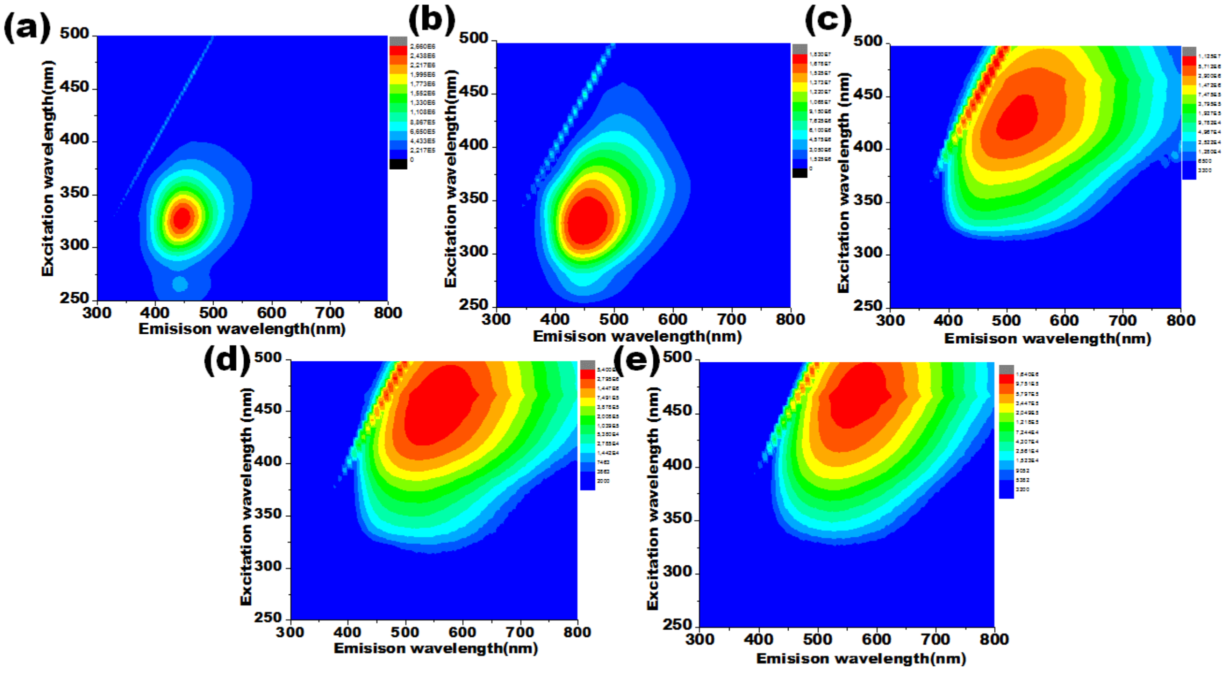

Fig. S4 Representative 2D excitation-emission contour map of carbon dots (a) CD1 (b) CD2 (c) CD3 (d) CD4 and (e) CD5.

 This journal is © The Royal Society of Chemistry 20xx

Please do not adjust margins

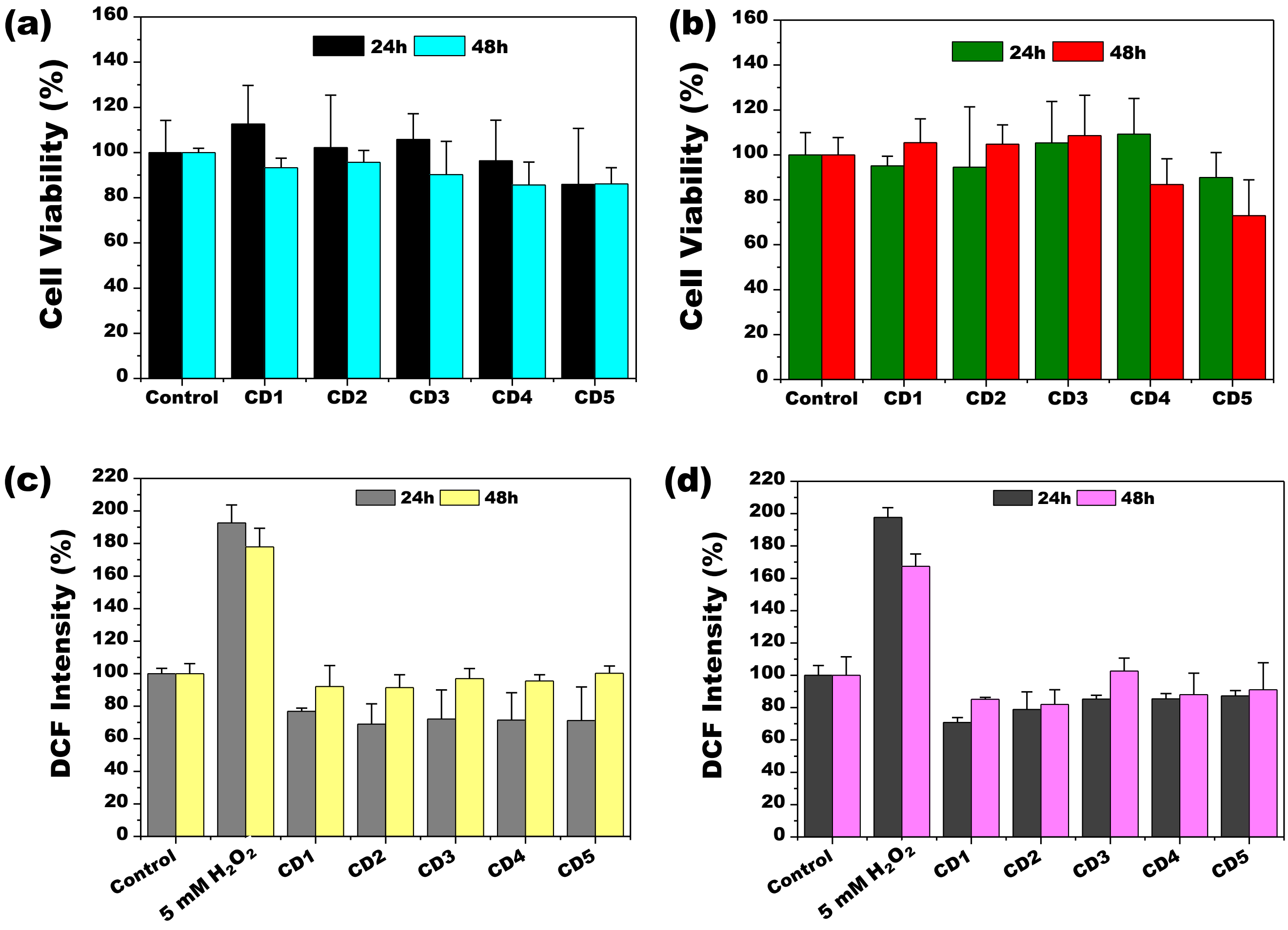

Fig. S5 Biocompatibility studies of carbon dots. Cellular effects of carbon dots in Caco-2 human colon carcinoma cells using WST-1 cell viability and H_2DCF-DA assay at 100 µg/mL (a&c) and 250 µg/mL (b&d). Error bars depict ± standard error of the mean for *n*=3.

Please do not adjust margins

Please do not adjust margins

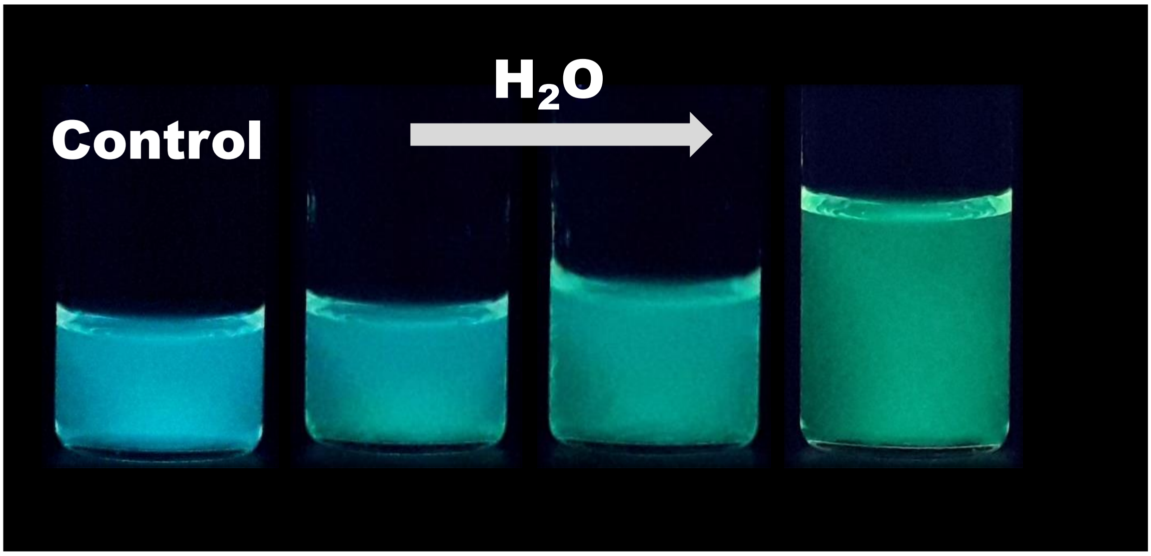

Fig. S6 Visual colorimetric changes in the emission of CD1 in THF upon addition of water under UV light (λ_{max}-365 nm).

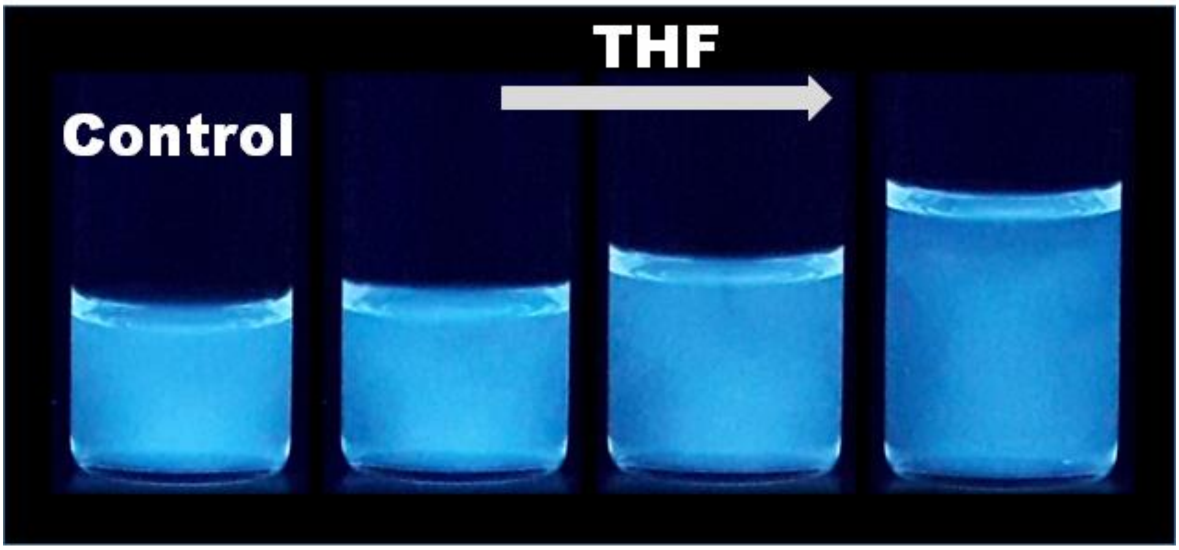

Fig. S7 Visual colorimetric changes in the emission of CD1 in THF upon addition of pure THF under UV light (λ_{max}-365 nm).

This journal is © The Royal Society of Chemistry 20xx

Please do not adjust margins

Please do not adjust margins

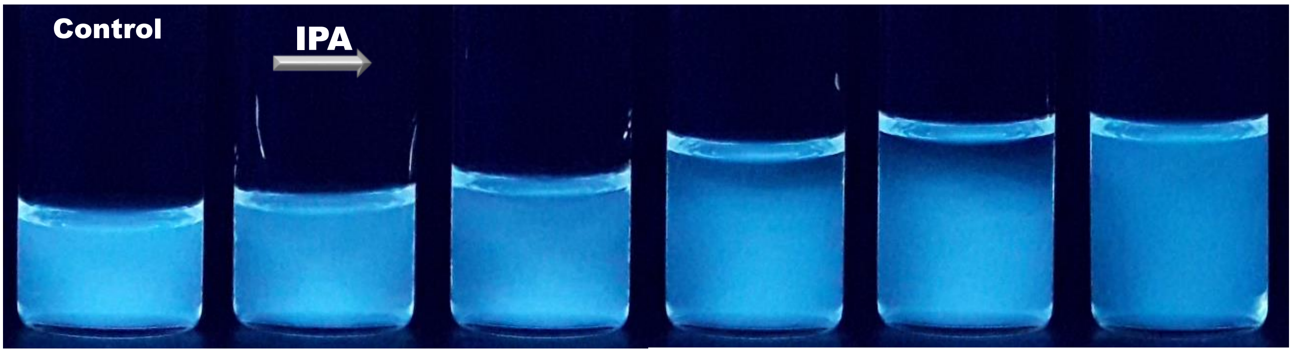

Fig. S8 Selective water sensing performance of CD1/THF. Photograph under UV light (λ_{max-}365 nm) showing the carbon dots in THF (2 mL) and subsequent addition of IPA (2 mL). The observed results indicated that there is no significant chromatic transition.

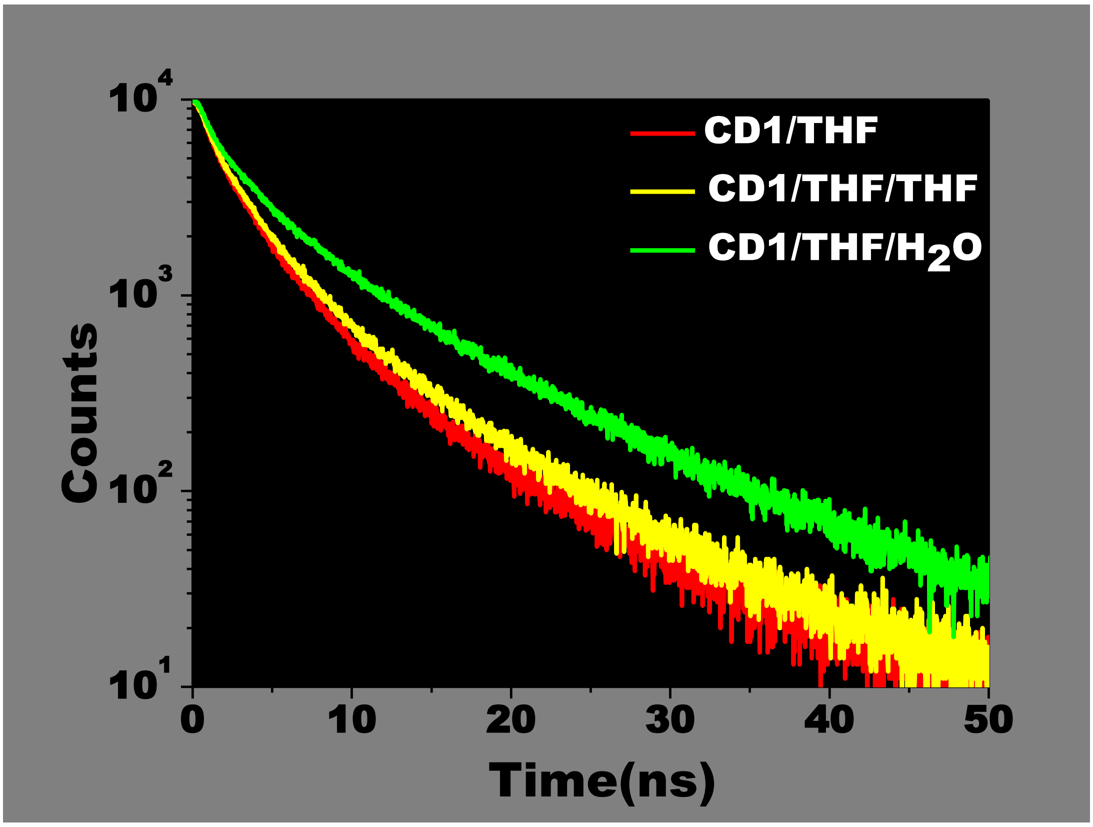

Fig. S9 Time-resolved photoluminescence decay curves.

Please do not adjust margins

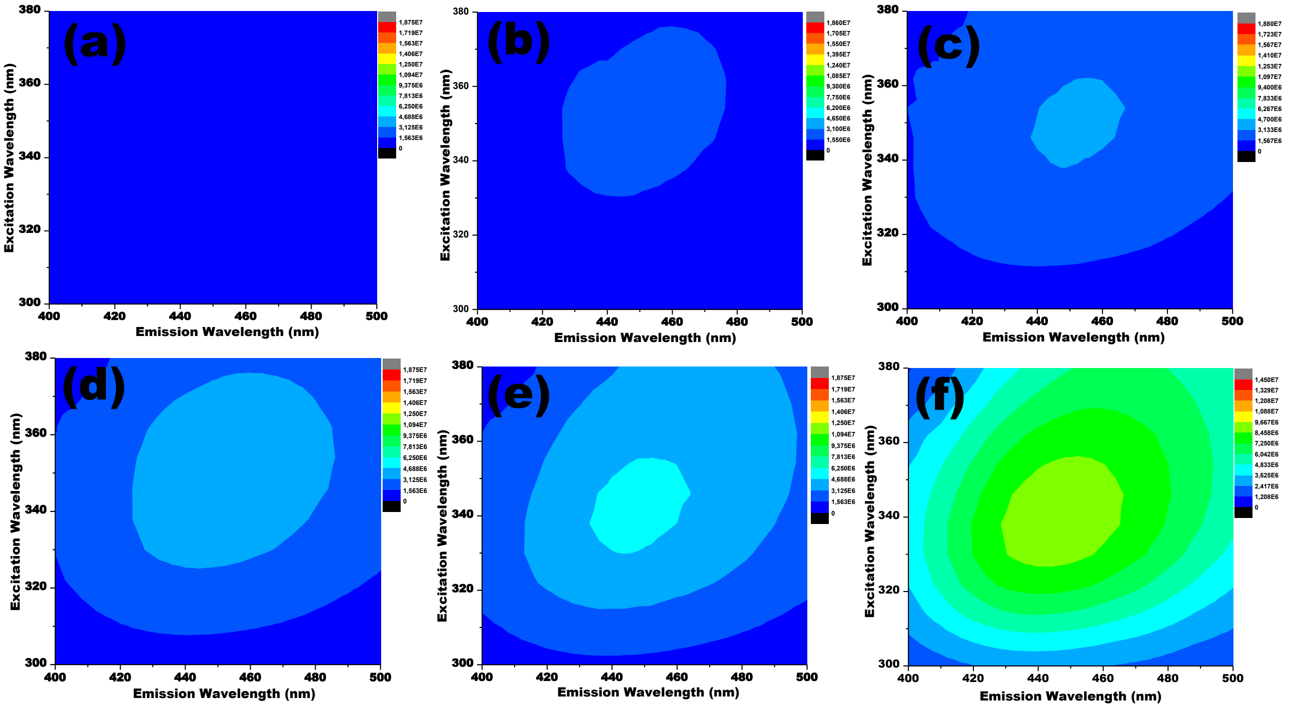

Fig. S10 Excitation-emission contour maps of carbon dots in (a) IPA and (b-f) hydrated IPA. (b) 0.2%, v/v (c) 0.6%, v/v (d) 1.0%, v/v (e) 3.0%, v/v and (f) 5.0%, v/v.

This journal is © The Royal Society of Chemistry 20xx

Please do not adjust margins

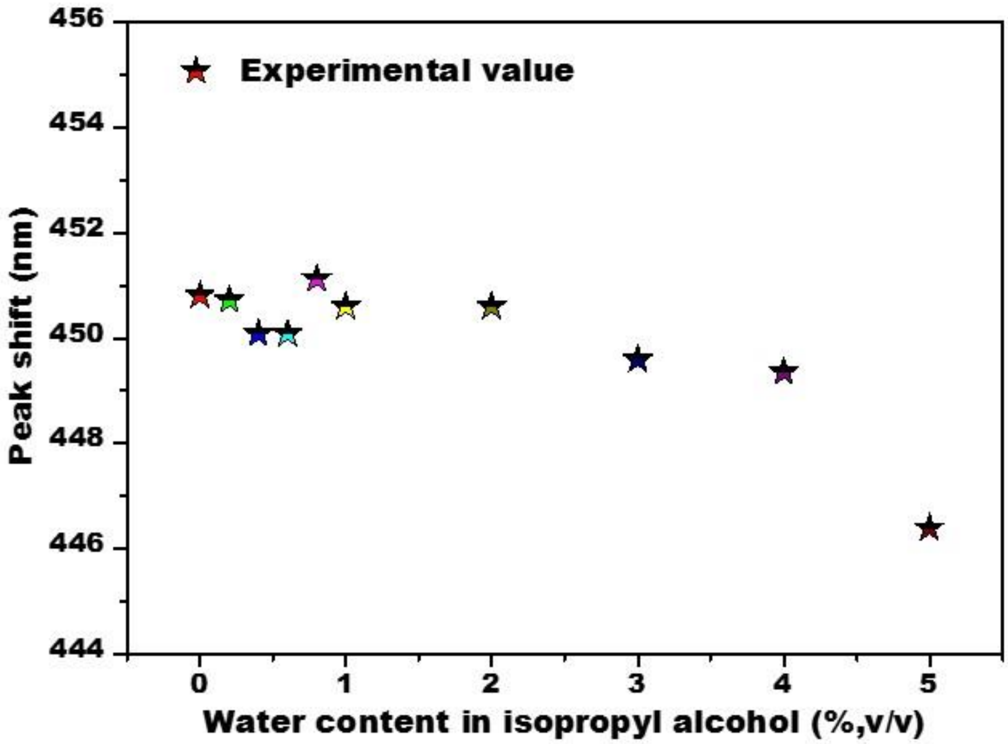

Fig. S11 Colorimetric sensing performance of hydrated Isopropyl alcohol. PL emission spectra (λ_{ex}-330 nm) peak shift vs water content (0.2 % to 5.0% v/v).

Please do not adjust margins

Supplementary Table 1: Comparison of the carbon dot based photoluminescent water sensor with other works.

Material	Change in the spectral position	Visual demonstration of photo/video	Type of solvent	Response time	Solvent specific response	Ref
2D oxamato-based manganese(II)-copper(II) mixed-metal–organic framework	Increased/Shift (586 to 544 nm)	No	Toluene	Greater than 60 minutes	No	1
Mg^{2+} metal–organic framework	Increased/Shift	No	THF, EtOH and ACN	1–2 minutes	No	2
4′-N, N-dimethylamino-4-methylacryloylamino chalcone (DMC)	Decreased	No	ethanol, acetone, and THF	less than 90s	No	3
Europium Coordination Compound $(Eu(DAF)_2(NO_3)_3)$	Decreased	No	acetonitrile	-	No	4
Copper nanoclusters	Decreased	No	DMSO, DMF, THF, Acetonitrile	20 min	No	5
Oxoporphyrinogens	Absorption Spectra	No	THF	-	No	6
Tetraphenylethene (TPE) derivatives	Increased	Yes	THF and dioxane	-	No	7
Coumarin-decorated Schiff base	Increased/Shift	No	DMSO	-	No	8
Flexible Indium(III) Metal–Organic Polyhedra Materials	Decreased	Yes	acetonitrile	-	No	9
Zn-MOF (metal–organic framework, $Zn(hpi2cf)(DMF)(H_2O)$)	Shift/Increased	yes	THF, MeOH, Acetone, ACN and DMF	Instantly	No	10
Carbon dots	Shift/Decreased -Aprotic solvents Increased-Protic solvents	yes	THF, IPA, ACN, Acetone and ButOH.	Instantly	Yes	Current work

This journal is © The Royal Society of Chemistry 20xx

Please do not adjust margins

References:

1. J. Ferrando-Soria, H. Khajavi, P. Serra-Crespo, J. Gascon, F. Kapteijn, M. Julve, F. Lloret, J. Pasán, C. Ruiz-Pérez, Y. Journaux and E. Pardo, *Adv. Mater.,* 2012, **24**, 5625.
2. A. Douvali, A. C. Tsipis, S. V. Eliseeva, S. Petoud, G. S. Papaefstathiou, C. D. Malliakas, I. Papadas, G. S. Armatas, I. Margiolaki, M. G. Kanatzidis, T. Lazarides and M. J. Manos, *Angew. Chem. Int. Ed.,* 2015, **54**, 1651.
3. X. Y. Wang, C. G. Niu, L. Y. Hu, D. W. Huang, S. Q. Wu, L. Zhang, X. J. Wen and G. M. Zeng, *Sens. Actuators B Chem.,* 2017, **243**, 1046.
4. L. Song, Y. W. Wu, W. X. Chai, Y. S. Tao, C. Jiang and Q. H. Wang, *Eur. J. Inorg. Chem.,* 2015, **13**, 2264.
5. Y. Huang, W. Liu, H. Feng, Y. Ye, C. Tang, H. Ao, M. Zhao, G. Chen, J. Chen and Z. Qian, *Anal. Chem.,* 2016, **88**, 7429.
6. S. Ishihara, J. Labuta, T. Šikorský, J. V. Burda, N. Okamoto, H. Abe, K. Ariga and J. P. Hill, *Chem. Commun.,* 2012, **48**, 3933.
7. Y. Zhang, D. Li, Y. Li and J. Yu, *Chem. Sci.,* 2014, **5**, 2710.
8. W. Y. Kim, H. Shi, H. S. Jung, D. Cho, P. Verwilst, J. Y. Lee and J. S. Kim, *Chem. Commun.,* 2016, **52**, 8675.
9. X. Du, R. Fan, L. Qiang, Y. Song, K. Xing, W. Chen, P. Wang and Y. Yang, *Inorg. Chem.,* 2017, **56**, 3429.
10. L. Chen, J. W. Ye, H. P. Wang, M. Pan, S. Y. Yin, Z. W. Wei, L. Y. Zhang, K. Wu, Y. N. Fan and C. Y. Su, *Nat. Commun.,* 2017, **8**, 15985.

This journal is © The Royal Society of Chemistry 20xx

Please do not adjust margins
